# Antiseptic Dressing of Prof. Lister

**Published:** 1878-09

**Authors:** 


### The Antiseptic Dressing of Prof. Lister.

A large audience assembled to hear the distinguished English professor read a paper on his antiseptic dressing. It occupied more than an hour and was attentively listened to.

In all wounds, said Lister, there are two things to combat, 1st, the *ordinary inflammation.* 2d, the *suppuration caused by putrefaction.* A recent wound does not suppurate for twenty-four hours. Proud flesh or granulations always precede suppuration; this is a point of great moment. In certain cases of grafting practice by the method of Reverdi, we see a union by the first intention though granulations are present. This proves that the granulations in wounds have no tendency to form pus. As soon as a granulating surface is relieved from all causes of irritation it ceases to form pus. A recent wound dressed with phenic oil excites a flow of pus more or less abundant. But if between the antiseptic liquid and the wound we place an isolating substance which protects the wound against the irritation of the antiseptic, usually there is no suppuration, and no granulations are developed. At the end of some weeks a cicatrice is formed without a purulent flow. The protector or protective substance which he employs as all know is a species of silk gauze varnished with copal. It is not as yet sufficiently perfect to absolutely prevent suppuration of the granulating surface; nevertheless it greatly diminishes it. There is often danger he said in using too large a piece of the protector. The isolating layer excludes the irritant action of the antiseptic; but it is necessary that this extend beyond the gauze on all sides.

Water dissolves at the most 1-15 of its volume of phenic acid; this solution is very caustic to the tongue. A solution of phenic oil of 1-10 is less irritant than an aqueous solution of 1-20. Finally, phenic acid combined with resin in the proportion of 1 to 5 is still better tolerated.

For a permanent dressing we should employ a substace which has a great affinity for phenic acid. Now a resin has the advantage of not being soluble in water and does not commingle with the drainage which comes from wounds. The object of the "*Macintosh*" or impermeable tissue placed between the two last folds of the gauze is to completely prevent the drainage from soaking through the gauze, and to conduct it to the margins. Phenic is not the only acid used by Lister. Boric acid, which is milder, produces good results for superficial wounds and old ulcers; but in dressing with borac acid the protection has the disadvantage of removing the new epidermis which forms on the surface. A solution of zinc chloride of 1-12 he finds very advantageous in the mouth. A single application of it prevents putrefaction on the surface of the wound however numerous the causes of suppuration may be, as after extirpation of the tongue.

M. Desprès replied briefly to Prof. Lister and said that air does not exercise an evil influence on wounds. The studies of M. Bouisson on ventilation had very clearly shown this. As to wounds of the mouth, if let alone they nearly always healed without complications. He complained that Lister did not make sufficient account of immovability in wounds, and he (M. Desprès)

does not admit either that the opening of a congestive abscess even with antiseptic dressing constitutes a good practice.

Prof. Lister replied that the wounds of the mouth healed without treatment because the saliva constitutes a natural antiseptic and does not irritate the wound; but the healing is much more rapid after cauterization with the solution of zinc. On the other hand it is quite certain that an abscess by congestion often produces fever and a grave general condition. The opening of the abscess with antiseptic dressing is followed by great alleviation. He does not though pretend to cure every patient attacked with ossifluent abscess.—*Progrès Medicale.*—*Cincinnati Lancet.*

